# Leptin Modulates Norepinephrine-Mediated Melatonin Synthesis in Cultured Rat Pineal Gland

**DOI:** 10.1155/2013/546516

**Published:** 2013-07-01

**Authors:** Rodrigo Antonio Peliciari-Garcia, Jéssica Andrade-Silva, José Cipolla-Neto, Carla Roberta de Oliveira Carvalho

**Affiliations:** Department of Physiology and Biophysics, Institute of Biomedical Sciences, University of São Paulo, Avenue Professor Lineu Prestes 1524, Room 118, 05508-900 São Paulo, SP, Brazil

## Abstract

Pineal melatonin synthesis can be modulated by many peptides, including insulin. Because melatonin appears to alter leptin synthesis, in this work we aimed to investigate whether leptin would have a role on norepinephrine- (NE-)mediated melatonin synthesis in cultured rat pineal glands. According to our data, cultured rat pineal glands express leptin receptor isoform b (*Ob-Rb*). Pineal expression of *Ob-Rb* mRNA was also observed *in vivo*. Administration of leptin (1 nM) associated with NE (1 *µ*M) reduced melatonin content as well as arylalkylamine-N-acetyl transferase (AANAT) activity and expression in cultured pineal glands. Leptin treatment per se induced the expression of STAT3 in cultured pineal glands, but STAT3 does not participate in the leptin modulation of NE-mediated pineal melatonin synthesis. In addition, the expression of inducible cAMP early repressor (ICER) was further induced by leptin challenge when associated with NE. In conclusion, leptin inhibition of pineal melatonin synthesis appears to be mediated by a reduction in AANAT activity and expression as well as by increased expression of *Icer* mRNA. Peptidergic signaling within the pineal gland appears to be one of the most important signals which modulates melatonin synthesis; leptin, as a member of this system, is not an exception.

## 1. Introduction

 During the night, the mammalian pineal gland, a neuroendocrine organ, synthesizes and releases its main product, melatonin [1]. The production of melatonin is mainly regulated by norepinephrine (NE) release in the perivascular space of the gland during the dark period [2] and follows the photoperiod duration. Therefore, melatonin presents both daily and seasonal rhythmic patterns of production throughout the year [3–5].

The interaction of NE with adrenergic receptors *β*_1_ and *α*_1_ triggers pineal hormone synthesis by activation of downstream pathways of the adrenergic receptors [6–8]. These activations induce transcription of tryptophan hydroxylase (TPOH) and arylalkylamine-N-acetyl transferase (AANAT), a key enzyme in the synthesis of melatonin. The last step of synthesis consists of the conversion of N-acetylserotonin to melatonin by the hydroxyindole-O-methyltransferase [9]. There are at least three major mechanisms that modulate the transcription and/or stability of AANAT and therefore the synthesis of the pineal hormone. The first is the phosphorylation of CREB, a positive signal for *Aanat* expression. The second is the phosphorylation of AANAT and the association with 14-3-3, which protects AANAT against proteasomal proteolysis [10, 11]. The third is the induction of the inducible cAMP early repressor (ICER), which directly inhibits transcription of the *Aanat* gene [12–14].

Although regulated by NE, melatonin synthesis can also be modulated by many other factors, including vasoactive intestinal polypeptide (VIP), pituitary adenylate cyclase-activating peptide (PACAP), neuropeptide Y (NPY) [15], glutamate [16], angiotensin [17], and insulin [18, 19].

The fact that a glycemia-sensitive hormone such as insulin is able to modulate rat pineal melatonin synthesis highlights feeding as an important factor in the regulation of melatonin production. Therefore, leptin, which is synthesized by adipocytes and is related to the regulation of body weight homeostasis and satiety, should be an important candidate to be considered as a possible modulator of melatonin synthesis. However, there are a few reports in the literature regarding leptin action in the mammalian pineal gland, though it has been demonstrated that in seasonal breeding ewes leptin presents a dual effect on melatonin synthesis, stimulating it during short days and suppressing it during long ones [20–22].

Conversely, the effects of melatonin on leptin secretion have been previously demonstrated [23–28], although there is currently not a clear consensus in the literature regarding the effect of pineal gland hormone on mediation of leptin production.

Despite that leptin receptor mRNA has already been characterized in the bovine pineal gland [29], neither the presence nor the effect of leptin within the rat pineal gland has been investigated. Thus, the aim of the present study was to investigate whether leptin could modulate NE-mediated melatonin synthesis in cultured rat pineal gland. 

## 2. Material and Methods

### 2.1. Animals

Male Wistar rats weighing 150–180 g were obtained from the Institute of Biomedical Sciences, University of São Paulo, São Paulo, Brazil. The animals were kept under a 12 h/12 h light/dark (LD) cycle (lights on at 06:00), in a temperature controlled room (21 ± 2°C), with food and water *ad libitum*. Ethics approval was granted by the Committee of Ethics in Animal Experimentation of the Institute of Biomedical Sciences, University of São Paulo, São Paulo, Brazil.

### 2.2. Experimental Design

Pineal glands were cultured as previously described [18]. Briefly, after decapitation rat pineal glands were isolated at the end of the light phase and immediately placed in ice-cold BGJb (Fitton-Jackson Modification) medium with phenol red, modified by the addition of bovine serum albumin (BSA; 1 mg/mL), 2 mM glutamine, 0.1 mg/mL ascorbic acid, and penicillin (100 U/mL)—streptomycin (100 *μ*g/mL) (Gibco, Grand Island, NY, USA). Pineal glands, 2 glands/well, were incubated at 37°C, 95% O_2_, 5% CO_2_ in modified BGJb medium in 24-well plates (200 *μ*L of medium/well) for 48 h before any treatment (the medium was changed every 24 h interval). After 48 h, the glands were divided into the following experimental groups: control (cultured glands without treatments), NE (1 *μ*M), NE+leptin (Lep—100 pM; 1 nM, 10 nM, 100 nM, 1 *μ*M and 5 *μ*M, Sigma, St. Louis, MO, USA), and 1 nM of leptin alone was also used in specific experiments. 12 glands were used in each group, and the experiment was repeated 3 times. After 5 h of incubation the glands from each group were frozen on dry ice and kept at −80°C prior to subsequent analysis.

### 2.3. RNA Extraction, DNase Treatment, and Real-Time PCR

**Figure 1:**
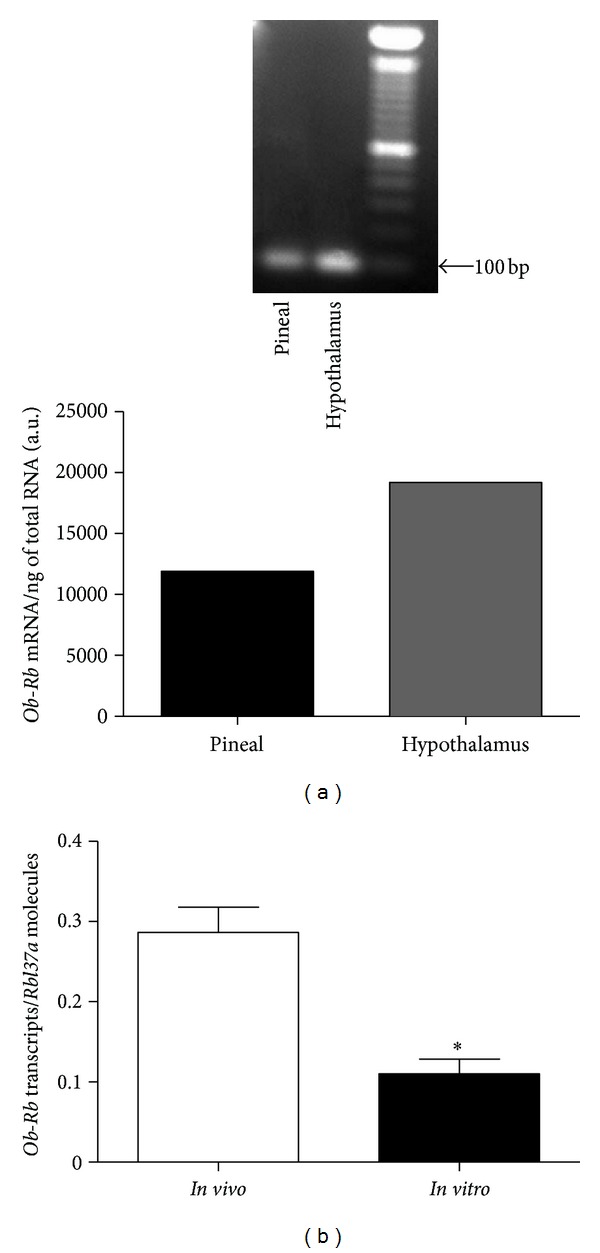
Expression of *Ob-Rb* mRNA in male rat pineal gland. (a) *In vivo* qualitative expression of *Ob-Rb *mRNA, pool of six (6) pineal glands harvested in the middle of the dark phase. Data presented as arbitrary units. (b) Real-Time RT PCR analysis of* in vivo* and *in vitro Ob-Rb *mRNA expression in the rat pineal gland. Data are reported as the number of transcripts per number of ribosomal protein L37a (*Rpl37a*) molecules. Student's *t*-test, unpaired, and two-tailed, *P < 0.05 versus *in vivo. n* = 6 glands/group and each experiment was repeated 3 times.

Total RNA was isolated from rat pineal glands using TRIzol (Invitrogen, Carlsbad, CA, USA) according to the manufacturer's instructions. DNase treatment was performed using Turbo DNA-*free* kit according to the kit's directions (Ambion, Austin, TX, USA). cDNA synthesis was performed using Super Script III Reverse Transcriptase (Invitrogen, Carlsbad, CA, USA) from 1 *μ*g of total RNA. 5 ng of the obtained cDNA was used in all qPCR assays, which were performed on the 7500HT Fast Real-Time PCR System, using Power SYBR Green (Applied Biosystems, Foster City, California, USA). Primer sequences for rat *Tpoh*, *Aanat,* and *Hiomt* have been previously published by our group [18], while specific primers assays for leptin receptor isoform b (*Ob-Rb*)*, Icer, *and* Rpl37a *were designed from rat sequences available in the GenBank and are presented in Table 1. Absolute qPCR quantification was performed using DNA standards preparation (number of molecules) for each investigated gene [30]. Real-Time PCR data are reported as the number of transcripts per number of ribosomal protein L37a (*Rpl37a*) molecules. Qualitative PCR results (showed only in Figure 1(a)), show the expression of *Ob-Rb* mRNA/ng of total RNA (arbitrary units) in a pool of 6 pineal glands. For all gene expression analysis, except Figure 1(a), it was used 6 glands/group, and all experiments were repeated 3 times.

### 2.4. Enzyme Activity

AANAT activity was measured by a radiometric assay [31, 32]. Briefly, 100 *μ*L of 0.1 M sodium phosphate buffer, pH 6.8, containing 40 mM tryptamine and [^3^H]-acetyl coenzyme A (2 mM, final specific activity = 4 mCi/mmol) was added to a microcentrifuge tube containing one gland kept at 4°C. All glands were sonicated and then incubated at 37°C for 20 min. The reaction product N-^3^[H]-acetyltryptamine was extracted with chloroform (1 mL) and presented as pmols/gland/h. 500 *μ*L samples were evaporated until dry in a scintillation vial and radioactivity was determined with a Beckman LS6500 *β* counter. 12 glands were used in each group, and the experiment was repeated 3 times.

### 2.5. Immunoblotting

Total protein extraction and immunoblotting (IB) were performed as previously described [19]. Briefly, pineal glands were homogenized in solubilization buffer (100 mM Tris, 1% SDS, 10 mM EDTA, 100 mM Na_2_P_2_O_7_, 100 mM NaF, 10 mM Na_2_VO_4_). 9 glands were used in each group, and the experiment was repeated 3 times. The homogenates were centrifuged at 17,530 g for 20 min at 4°C to remove insoluble material. Protein concentration in the supernatants was determined by the Bradford dye method using Bio-Rad reagent. Proteins were treated with Laemmli [33] sample buffer containing dithiothreitol and boiled for 5 min before being loaded into sodium dodecyl sulfate-polyacrylamide gel electrophoresis (SDS-PAGE) in a Bio-Rad miniature slab gel apparatus. Same concentration aliquots (50 *μ*g) were subjected to SDS-PAGE. Protein transfer from the gel to the nitrocellulose membrane was performed for 45 min at 15 V in a Bio-Rad semidry transfer apparatus. Nonspecific protein binding to the nitrocellulose was reduced by overnight incubation at 4°C in Odyssey blocking buffer (Li-Cor, Lincoln, NE, USA). Membranes were incubated overnight at 4°C with specific antibodies and then probed with specific fluorescence dye (IRDye Li-Cor, Lincoln, NE, USA) for 1 h at room temperature. The membranes were then scanned and the band intensities were quantified using Odyssey scan (Li-Cor, Lincoln, NE, USA). Reagents for SDS-PAGE, IB, and nitrocellulose membrane (0.45 *μ*m) were obtained from Bio-Rad (Hercules, CA, USA). Trizma, aprotinin, dithiothreitol, Triton X-100, glycerol, Tween 20, bovine serum albumin, and fraction V were obtained from Sigma (St. Louis, MO, USA). Antibodies against STAT3 and *α* Tubulin were purchased from Millipore (Billerica, MA, USA) and Santa Cruz Biotechnology Inc. (Santa Cruz, CA, USA), respectively.

### 2.6. Chromatography

Pineal glands (12 glands/group) or culture medium (6 wells/group = 2 glands/well) melatonin levels were measured by ultra high performance liquid chromatography (Dionex UHPLC Ultimate 3000) with electrochemical detection (ESA Coulochem III) and autosampler (WPS-3000TSL with sample thermostatting) running Chromeleon software and plotted as ng/gland or ng/well (200 *μ*L of medium). Melatonin was separated on an Acclaim RSLC 120 C18 column (2.2 *μ*m, 120 A, 100 × 2.1 mm). The chromatographic system was isocratically operated with the following mobile phase: 0.1 M sodium acetate, 0.1 M citric acid, 0.15 mM EDTA, 30% methanol, pH 3.7, and at a flow rate of 0.135 mL/min. The electrochemical detector potential was adjusted to + 750 mV in the guard cell and + 700 mV in the 5041 analytical cell. The elution time for melatonin was about 7 min. A solution of 0.1 M of perchloric acid, containing 0.02% EDTA and 0.02% sodium bisulfate was used to extract melatonin content. Pineal glands were sonicated in 120 *μ*L of this solution, and culture medium was treated 1 : 1 (v/v). Each experiment was repeated 3 times.

### 2.7. Statistical Analysis

The HPLC, enzyme activity assay, RT PCR, and protein expression data were plotted as the mean ± SEM. Student's *t*-test was used when appropriate. One-way ANOVA followed by Bonferroni's post hoc test was performed using GraphPad Prism (GraphPad Software version 5.01, San Diego, CA, USA).

## 3. Results

 The qualitative mRNA expression of *Ob-Rb *in the rat pineal gland is shown in Figure 1(a). Hypothalamus was used as a positive control, though no comparisons were made between tissues (data are presented as arbitrary units). The Real-Time PCR analysis of rat pineal glands revealed a reduction of 62% in the *in vitro* expression of *Ob-Rb* mRNA (0.11 ± 0.01) in comparison to the *in vivo *condition (measured here as a positive reference) (0.28 ± 0.03, *P* < 0.05) (Figure 1(b)).

**Figure 2:**
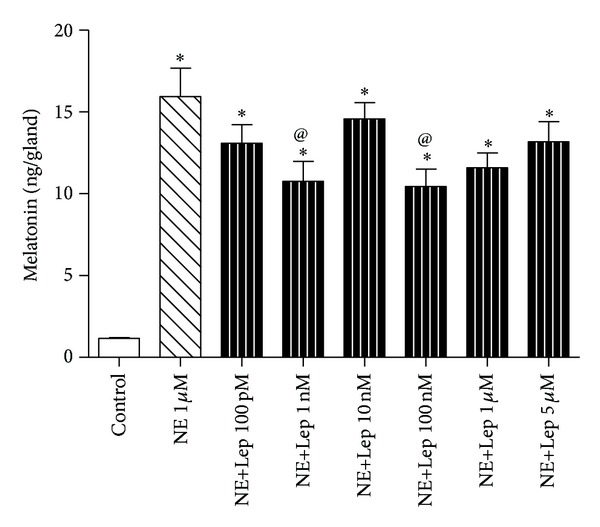
Leptin effect on NE-induced melatonin synthesis in cultured male rat pineal gland. The glands were challenged with different concentrations of leptin (Lep) (100 pM = 5 *μ*M) associated with norepinephrine (NE) (1 *μ*M). Melatonin values are expressed in ng/gland. One-way ANOVA, followed by Bonferroni's post hoc test. *P < 0.05 versus control, *P* < 0.05 versus NE 1 *μ*M. *n* = 12 glands/group and each experiment was repeated 3 times.

 Cultured pineal glands were challenged with varying doses of leptin associated with norepinephrine (1 *μ*M). Two distinct doses of leptin (1 nM and 100 nM) were able to reduce melatonin content by 33 and 35%, respectively, when compared to NE alone (10.75 ± 1.22 and 10.45 ± 1.05 versus 15.94 ± 1.73, *P* < 0.05). As both doses had similar responses, 1 nM was selected for use in the following experiments. All other leptin doses reduced melatonin content, but not to a statistically significant level. NE+Lep 10 nM presented a slight, though not significant, difference in comparison to other NE+Lep concentrations, but it also presented lower values than NE alone (Figure 2).

**Figure 3:**
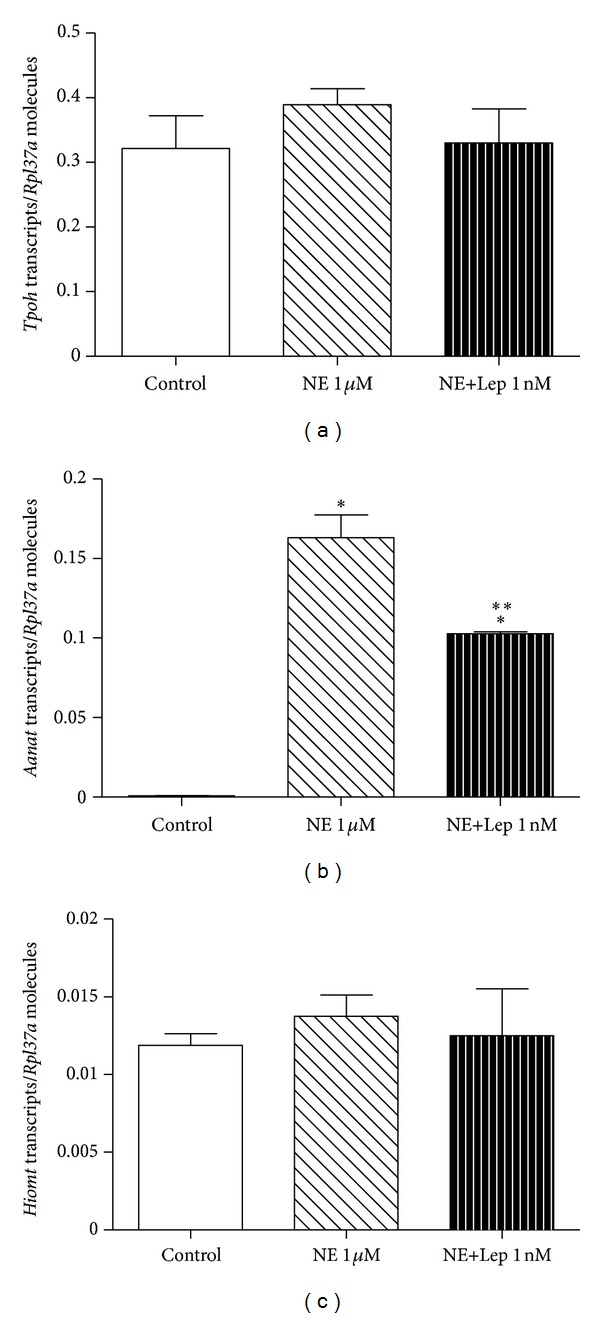
Leptin effect on *Tpoh, Aanat,* and *Hiomt* mRNA expression in cultured rat pineal gland. The glands were challenged with leptin (Lep 1 nM) associated with norepinephrine (NE 1 *μ*M). (a) Tryptophan hydroxylase (*Tpoh*) mRNA expression. (b) Arylalkylamine-N-acetyl transferase (*Aanat*) mRNA expression. (c) Hydroxyindole-O-methyltransferase (*Hiomt*) mRNA expression. One-way ANOVA, followed by Bonferroni's post hoc test. *P < 0.05 versus control. Data are reported as the number of transcripts per number of ribosomal protein L37a (*Rpl37a*) molecules. *n* = 6 glands/group and each experiment was repeated 3 times.

The expression of *Aanat* mRNA was reduced when challenged with 1 nM of leptin associated to NE in comparison to NE alone (0.10 ± 0.001 versus 0.16 ± 0.01, *P* < 0.05, reduction of 38%). As expected, a strict dependence on NE stimulus was observed in *Aanat* mRNA expression, indicated by a paucity of *Aanat* expression in the non-NE stimulated control group (0.16 ± 0.01 NE alone versus 0.0008 ± 0.0001 control, *P* < 0.05). The expressions of *Tpoh* and *Hiomt* were not affected by the leptin treatment (Figures 3(a), 3(b), and 3(c)).

**Figure 4:**
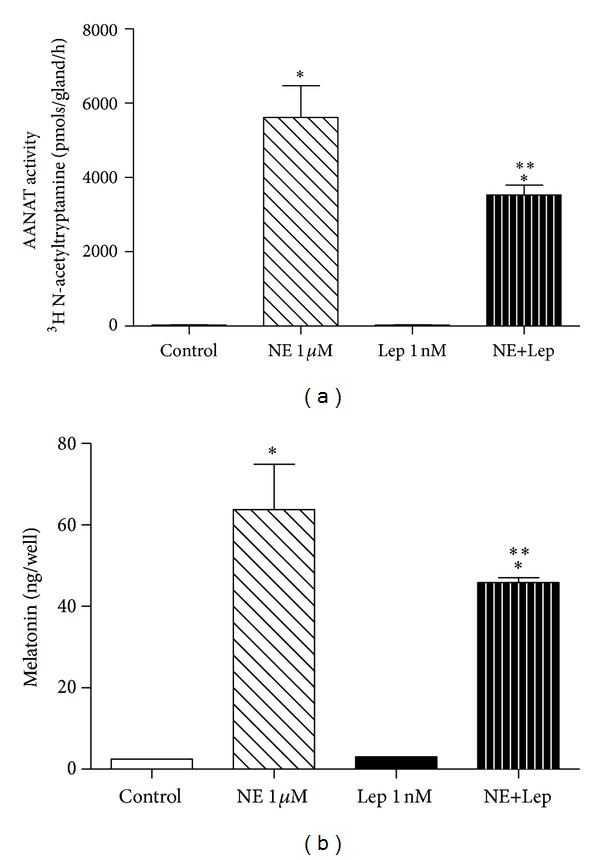
Leptin effect on AANAT activity and melatonin synthesis in cultured rat pineal gland. The glands were challenged with leptin (Lep 1 nM) alone and in association with norepinephrine (NE 1 *μ*M). (a) AANAT activity was determined using cultured pineal glands, values are expressed in pmols/gland/h. (b) Melatonin content was quantified from pineal gland culture medium, values are expressed as ng/well. One-way ANOVA, followed by Bonferroni's post hoc test. *P < 0.05 versus control, **P < 0.05 versus NE 1 *μ*M. *n* = 12 glands/group and each experiment was repeated 3 times.

The activity of AANAT was investigated under the same conditions, but the glands were also challenged with leptin alone (1 nM). AANAT activity was determined using the cultured glands, while melatonin content was quantified from the cultivation medium of the same experiment. Once again, reductions in AANAT activity (38%) and melatonin content (29%) were observed in the NE+Lep group in comparison to the NE group (3528 ± 267 versus 5615 ± 855 and 45.82 ± 1.20 versus 63.80 ± 11.08, *P* < 0.05, resp.) (Figures 4(a) and 4(b)). Leptin alone had no effect on AANAT activity or melatonin synthesis, while NE treatment per se induced a significant difference in both AANAT activity and melatonin synthesis when compared to the control group (5615 ± 855 versus 31.74 ± 6.01 and 63.80 ± 11.08 versus 2.5 ± 0.001, *P* < 0.05, resp.) (Figures 4(a) and 4(b)).

**Figure 5:**
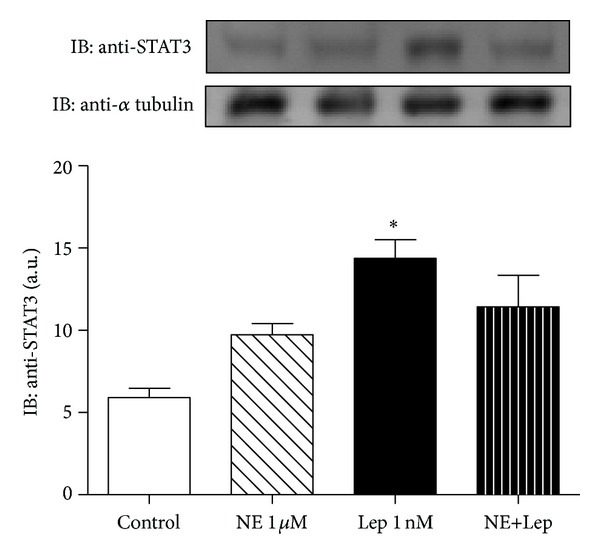
Leptin signaling in cultured rat pineal gland. The glands were challenged with leptin (Lep 1 nM) alone and in association with norepinephrine (NE 1 *μ*M). Immunoblotting (IB) was performed against STAT3, a downstream protein of leptin signaling. *α* Tubulin is showed as internal control; values are expressed as arbitrary units. One-way ANOVA, followed by Bonferroni's post hoc test. *P < 0.05 versus control. *n* = 9 glands/group and each experiment was repeated 3 times.

 Leptin challenge per se induced a 143% increase in the expression of STAT3 in cultured rat pineal glands when compared to the control group (14.38 ± 1.12 versus 5.90 ± 0.57, *P* < 0.05). Treatment with both NE and NE+Lep also increased STAT3 levels, though no difference in induction was observed between the two groups or even in comparison to control group (Figure 5).

**Figure 6:**
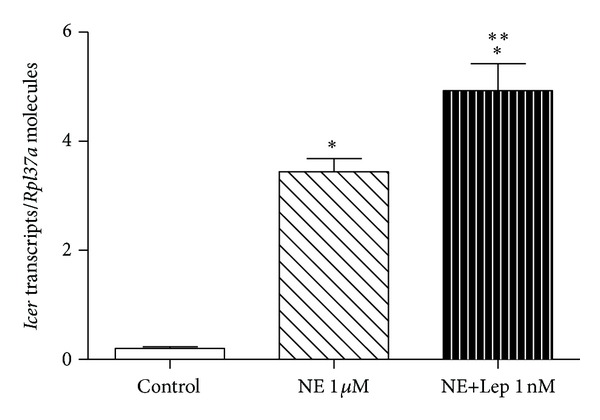
Leptin effect on *Icer *mRNA expression in cultured rat pineal gland. The glands were challenged with leptin (Lep 1 nM) associated with norepinephrine (NE 1 *μ*M). One-way ANOVA, followed by Bonferroni's post hoc test. *P < 0.05 versus control, **P < 0.05 versus NE 1 *μ*M. Data are reported as the number of transcripts per number of ribosomal protein L37a (*Rpl37a*) molecules. *n* = 6 glands/group and each experiment was repeated 3 times.

Finally, the expression of *Icer*, which directly inhibits the transcription of *Aanat*, was investigated in cultured pineal glands. Interestingly, the expression of *Icer* was further stimulated by leptin associated with NE than by NE alone (4.92 ± 0.49 versus 3.44 ± 0.24, *P* < 0.05, increase of 43%) (Figure 6).

## 4. Discussion

In this work, we aimed to investigate the effects of leptin on the modulation of melatonin synthesis in cultured rat pineal glands.

There are six isoforms of Ob-R, (a, b, c, d, e, and f), but only the full-length isoform *Ob-Rb*, which is mainly expressed in the hypothalamus, is able to fully transduce an activation signal into the cell [34, 35]. In our study, the *in vivo* and *in vitro* expressions of *Ob-Rb* mRNA in rat pineal glands were characterized. The general reduction of mRNA expression in pineal gland culture condition has been previously observed by our laboratory (unpublished data). However, the reduced expression of *Ob-Rb* mRNA observed *in vitro* in this study seems not to affect the ability of leptin to signal in cultivated pineal glands. The expression of *Ob-Rb* mRNA in the pineal gland corroborates with previous results from another mammalian pineal gland [29].

 The effect of leptin on pineal melatonin synthesis in seasonal breeding ewes has been previously investigated. According to melatonin content measurements from *in vitro* and *in vivo* experiments in these studies, leptin presents a dual effect, being able to both stimulate melatonin synthesis during short days and suppress it during long days [20–22]. In cultured rat pineal glands, leptin appears to inhibit production of the neurohormone by reducing the mRNA expression and activity of AANAT, an important enzyme involved in the melatonin synthesis cascade. The rhythmic expression of leptin, which peaks in the middle of the dark phase, was already demonstrated in rat [36]. Leptin does not appear to need to be in phase with melatonin to present an effect, as already demonstrated in diurnal animals (ewes), but in the rat, since both hormones peak during the night phase, this could refine the modulatory effect of leptin on melatonin synthesis.

In our experiments, 5 h of leptin challenge was able to induce the total amount of STAT3 expression. This increase in STAT3 might be responsible for the activation of the leptin signaling pathway within cultured rat pineal gland. At the same time, STAT3 appears to have no role in the modulation of melatonin synthesis induced by leptin, as there was no difference in melatonin synthesis when NE was administrated with or without leptin.

Interestingly, when challenged with leptin associated with NE, it was seen an increased induction of *Icer* mRNA expression. ICER is an important inhibitor of *Aanat* transcription and therefore a suppressor of melatonin synthesis. Its expression is triggered by the activation of noradrenergic *β* receptors [12–14]. Ho and colleagues (2007) demonstrated that ICER is only effective in repressing AANAT activity and melatonin synthesis in an acute manner when pinealocytes are exposed to higher levels of ICER than standard levels regularly induced by NE [37]. In our results NE+Lep also evoked higher expression of *Icer* mRNA in comparison to the standard NE induction levels. Therefore, according to our data it is possible to speculate that the increase in *Icer* mRNA might be an important factor for the reduced AANAT activity and melatonin synthesis observed in this study.

It has been demonstrated in the literature that melatonin acts in adipose tissue [38] and also can inhibit leptin synthesis [25–28], although contrary results have also been seen [23, 24]. According to our results, leptin can inhibit pineal melatonin synthesis. Therefore, an interrelationship between the synthesis of both hormones seems to exist. This mechanism might be important in pathological conditions such as obesity, where both leptin and melatonin levels are altered [39–41].

The pineal melatonin synthesis is under a complex regulation. Pineal gland receives signaling from many different systems (i.e., peptidergic signaling). These signals ensure the ability to tune melatonin synthesis, which mediates multiple biological processes in the organisms [42, 43]. Thus, it is not a surprise to find that leptin is also part of this complex system. In this first approach, we characterized the effect of leptin on NE-mediated melatonin synthesis in cultured rat pineal glands. How leptin cascade signaling affects *Icer* and AANAT activity and expression and how short or long days could alter the manner in which leptin signals to the rat or hamster (since hamsters are seasonal animals) pineal glands remain to be investigated.

 In summary, leptin challenge inhibits NE-mediated melatonin synthesis in cultured rat pineal gland. This inhibition seems to be caused by a reduction in AANAT activity and expression, additionally reinforced by an increase in *Icer* mRNA expression.

## Figures and Tables

**Table 1:** Primer sequences for rat qPCR assays.

Gene/GenBank accession number	Primer sequences	Amp. length
*Icer*/NM_001110860.1	Forward-5′-ACTCGAAAGCGGGAGCTGA-3′ Reverse-5′-ACATATTCTTTCTTCTTCCTGCGAC-3′	76

*Ob-Rb*/NM_012596.1	Forward-5′-CCAGCACAATCCAATCACTAGTG-3′ Reverse-5′-CGAATAGATGGATTATCGGGACA-3′	87

*Rpl37a/*NM_001108801	Forward-5′-TTGAAATCAGCCAGCACGC-3′ Reverse-5′-TGCCAACGGCTCGTCTCT-3′	74
